# Comparative Analysis of Clinical Outcomes in Patients With Type 2 Diabetes Mellitus on Monotherapy Versus Combination Therapy

**DOI:** 10.7759/cureus.104344

**Published:** 2026-02-26

**Authors:** Hariballav Mahapatra, Piyush Kumar Gupta, Ajo Paul, Amit Nampalliwar, Manju Bansal, Baijnath Das, K. Parameswaran Namboothiri

**Affiliations:** 1 Department of Diabetology, Sevayan Diabetes Centre, Puri, IND; 2 Department of Community Medicine, Geetanjali Institute of Medical Sciences, Jaipur, IND; 3 Department of Community Medicine, Sree Narayana Institute of Medical Sciences, Ernakulam, IND; 4 Department of Roganidan and Vikriti Vigyan (Pathology), Government Ayurved College and Hospital, Bilaspur, IND; 5 Department of Medicine, Dr. Rajendra Prasad Government Medical College, Tanda, IND; 6 Department of Medical Laboratory Technique, College of Paramedical Sciences, Teerthanker Mahaveer University, Moradabad, IND; 7 Department of Panchakarma, Amrita School of Ayurveda, Amrita Vishwa Vidyapeetham, Amritapuri, IND

**Keywords:** cardiovascular outcomes, combination therapy, glycemic control, monotherapy, type 2 diabetes mellitus

## Abstract

Type 2 diabetes mellitus (T2DM) is a highly prevalent metabolic disorder associated with substantial microvascular and macrovascular morbidity, necessitating effective and durable therapeutic strategies. Progressive pathophysiology involving insulin resistance, beta-cell dysfunction, and multisystem metabolic dysregulation limits the long-term effectiveness of single-agent therapy in many patients. This narrative review aims to compare clinical outcomes associated with antidiabetic monotherapy and combination therapy in individuals with T2DM, with emphasis on glycemic control, cardiovascular outcomes, metabolic effects, safety, adherence, and cost considerations. A comprehensive literature search of major electronic databases was performed to identify relevant studies published between 2015 and 2025, including randomised controlled trials, observational studies, real-world analyses, and systematic reviews involving adult T2DM populations. Evidence indicates that monotherapy remains appropriate in early disease stages and in patients with modest hyperglycemia, offering simplicity and favourable tolerability. Combination therapy demonstrates superior and more durable glycemic control through complementary mechanisms of action and is associated with improved cardiovascular and metabolic outcomes, particularly when agents with cardioprotective and weight-modifying properties are included. Safety profiles vary across therapeutic classes, with combination regimens requiring individualised selection to minimise adverse events and optimise adherence. Economic analyses suggest that higher initial costs for combination therapy may be offset by reduced complications and healthcare utilisation. Individualised treatment strategies integrating timely combination therapy support sustained metabolic control and improved long-term outcomes in T2DM.

## Introduction and background

Type 2 diabetes mellitus (T2DM) is a prevalent metabolic disorder associated with substantial microvascular and macrovascular complications [1,2]. Rising prevalence across diverse populations reflects demographic ageing, increasing obesity, reduced physical activity, and changing dietary patterns [3]. The clinical and economic implications of long-term glycemic control further emphasise the importance of effective therapeutic strategies in T2DM management [4]. Progressive pathophysiology involving insulin resistance, impaired beta-cell function, excessive hepatic glucose production, altered incretin signalling, and dysregulated glucagon secretion contributes to chronic hyperglycemia and declining durability of single-agent therapy over time [5,6]. This progressive nature necessitates therapeutic strategies capable of targeting multiple metabolic defects [7].

Pharmacologic options for T2DM have expanded considerably, encompassing agents with distinct mechanisms of action [8]. Metformin remains widely recommended as initial therapy based on established efficacy and safety [9]. Additional classes, including sulfonylureas, thiazolidinediones, dipeptidyl peptidase-4 inhibitors, sodium-glucose cotransporter-2 (SGLT2) inhibitors, glucagon-like peptide-1 (GLP-1) receptor agonists, insulin-based regimens, and dual incretin receptor agonists, allow the targeting of complementary metabolic pathways [10,11]. Monotherapy may achieve glycemic control in early disease with moderate hyperglycemia, whereas combination therapy provides additive or synergistic effects by addressing multiple components of the underlying pathophysiology [11,12].

Clinical decision-making regarding therapy initiation and intensification requires the consideration of glycemic status, disease duration, cardiovascular and renal comorbidities, body weight, hypoglycemia risk, tolerability, and cost [13]. Delayed intensification and therapeutic inertia contribute to prolonged inadequate glycemic control in practice [14]. Fixed-dose combinations and early dual-agent strategies have been proposed to improve treatment durability and simplify regimens [15]. Despite evolving treatment paradigms, comparative evaluation of monotherapy and combination therapy across long-term cardiovascular outcomes, safety, adherence, and cost remains inconsistently reported [16,17]. Variability in guideline recommendations reflects ongoing uncertainty regarding optimal timing and sequencing of therapy [18]. The increasing emphasis on outcome-driven management further underscores the need for integrated comparison beyond short-term glycemic endpoints [19].

Clinical outcomes differ substantially across drug classes, and the term combination therapy in this review primarily refers to metformin-based dual regimens and combinations incorporating SGLT2 inhibitors or GLP-1 receptor agonists. This review provides a structured comparison of monotherapy and combination therapy in T2DM, focusing on glycemic durability, cardiometabolic outcomes, safety, adherence, and cost to inform outcome-oriented clinical decision-making.

Objectives of the review

This narrative review aims to synthesise and critically appraise existing evidence comparing clinical outcomes of monotherapy and combination therapy in patients with T2DM. Focus is placed on glycemic efficacy, cardiovascular and metabolic outcomes, safety considerations, adherence, and cost-related implications relevant to contemporary clinical practice.

Methodology

Search Strategy

An extensive literature search was performed in leading electronic databases, including PubMed, Scopus, Web of Science, and Google Scholar, to identify relevant studies published between 2015 and 2025. Controlled vocabulary (MeSH terms) and free-text keywords were combined using Boolean operators. A representative PubMed search strategy was as follows: (“type 2 diabetes mellitus” OR “T2DM”) AND (“monotherapy” OR “single-agent therapy”) AND (“combination therapy” OR “dual therapy” OR “intensification”) AND (“glycemic control” OR “cardiovascular outcomes” OR “metabolic outcomes” OR “safety” OR “medication possession ratio” OR “proportion of days covered” OR “treatment persistence” OR “discontinuation rate” OR “direct medical cost” OR “total healthcare cost” OR “hospitalisation cost” OR “cost-effectiveness” OR “budget impact”). Filters were applied for human studies and English-language publications within the specified timeframe. Similar structured search strings were adapted for other databases. Manual screening of reference lists of eligible studies and relevant reviews was conducted to identify additional studies meeting the inclusion criteria.

Eligibility Criteria

Peer-reviewed studies published in English and involving human participants were considered to ensure clinical applicability. The eligibility criteria included randomised controlled trials, observational studies, cohort studies, real-world evidence studies, and systematic reviews directly comparing monotherapy and combination therapy in adult patients with T2DM. Included studies reported measurable outcomes related to glycemic control, cardiovascular or renal outcomes, metabolic parameters, adverse events, treatment adherence, or economic endpoints. Adherence outcomes were required to include quantifiable metrics such as medication possession ratio (MPR), proportion of days covered (PDC), persistence duration, or discontinuation rate. Economic outcomes were required to report measurable endpoints, including direct medical costs, total healthcare costs, hospitalisation costs, cost-effectiveness ratios, or budget impact analyses. Studies lacking quantifiable adherence or economic data were excluded from those respective analyses.

Study Selection Process

Study selection was conducted in two stages: initial screening of titles and abstracts followed by full-text review. Screening was performed independently by two reviewers, and disagreements were resolved through discussion and consensus.

Quality Assessment

Methodological quality assessment was performed using validated tools appropriate to the study design. Randomised controlled trials were evaluated using the Cochrane Risk of Bias tool, while observational studies were assessed using the Newcastle-Ottawa Scale. Studies judged to have a high risk of bias or insufficient methodological quality were excluded from the final synthesis.

Therapy Classification and Definitions

For consistency of reporting, treatment strategies were categorised by regimen type and drug class. Monotherapy was defined as the use of a single glucose-lowering agent, including metformin, sulfonylureas, thiazolidinediones, dipeptidyl peptidase-4 inhibitors, SGLT2 inhibitors, GLP-1 receptor agonists, insulin, or dual incretin receptor agonists. Combination therapy was defined as concurrent use of two or more glucose-lowering agents, including metformin-based dual therapy (e.g., metformin plus sulfonylurea, metformin plus dipeptidyl peptidase-4 inhibitor, metformin plus SGLT2 inhibitor, metformin plus GLP-1 receptor agonist), SGLT2 inhibitor plus GLP-1 receptor agonist combinations, and fixed-dose versus loose-dose regimens when reported. Outcomes were interpreted according to the specific drug classes included in each regimen rather than assuming uniform effects across all combination strategies.

Data Extraction and Synthesis

Data extraction was conducted independently by two reviewers using a standardised data collection framework capturing study design, population characteristics, intervention details, comparator groups, outcome measures, and duration of follow-up. For adherence outcomes, extracted variables included MPR, PDC, persistence rates, discontinuation rates, and time horizons. For economic outcomes, extracted variables included direct medical costs, total healthcare expenditure, hospitalisation rates with associated costs, and reported budget impact estimates. Findings were synthesised narratively, with outcome reporting structured around measurable endpoints and specified timeframes rather than general economic or behavioural interpretations.

## Review

Pathophysiology of T2DM and therapeutic targets

T2DM is characterised by progressive insulin resistance and declining pancreatic beta-cell function, leading to chronic hyperglycemia and disease progression [20]. Insulin resistance in the skeletal muscle, adipose tissue, and liver reduces peripheral glucose uptake and increases hepatic glucose production [18]. Progressive beta-cell dysfunction further impairs insulin secretion, contributing to worsening glycemic control over time [21,22]. Additional contributors include dysregulated glucagon secretion, impaired incretin response, and increased renal glucose reabsorption, which collectively amplify hyperglycemia and contribute to the limited durability of single-pathway therapies [7,23,24].

Monotherapy typically targets one dominant defect and may become insufficient as the disease progresses [20]. Combination therapy targets complementary mechanisms simultaneously, providing a mechanistic rationale for improved and more sustained glycemic control [25,26]. Table 1 summarises major pathophysiological defects in T2DM and corresponding therapeutic targets.

**Table 1:** Major pathophysiological mechanisms in T2DM and associated therapeutic targets Met: metformin; TZD: thiazolidinediones; SU: sulfonylureas; GLP-1RA: glucagon-like peptide-1 receptor agonists; DPP-4i: dipeptidyl peptidase-4 inhibitors; SGLT2i: sodium-glucose cotransporter 2 inhibitors

Pathophysiological defect	Primary metabolic effect	Therapeutic target	Drug class examples	Clinical impact	References
Insulin resistance	Reduced peripheral glucose uptake	Improved insulin sensitivity	Met, TZD	Lower fasting glucose	[27]
Beta-cell dysfunction	Impaired insulin secretion	Enhanced insulin release	SU, GLP-1RA, DPP-4i	Improved postprandial control	[19]
Excess hepatic glucose output	Increased gluconeogenesis	Suppressed hepatic glucose production	Met	Reduced fasting hyperglycemia	[22]
Glucagon dysregulation	Elevated glucagon levels	Glucagon suppression	GLP-1RA, DPP-4i	Reduced hepatic glucose output	[15]
Increased renal glucose reabsorption	Persistent hyperglycemia	Urinary glucose excretion	SGLT2i	Glucose-lowering with weight benefit	[24]

Monotherapy and rationale for combination therapy

Antidiabetic monotherapy remains an established approach in T2DM, particularly in early disease stages or in patients with modest hyperglycemia. Initial agent selection is guided by disease severity, metabolic profile, comorbidities, and safety considerations [28]. Monotherapy targets a dominant pathophysiological defect and offers a simpler regimen that is often well tolerated in early disease [6]. Metformin is widely recommended as first-line therapy due to its effects on hepatic gluconeogenesis, peripheral insulin sensitivity, and glucose utilisation [25,29]. Other agents may be used as monotherapy depending on clinical context, including sulfonylureas, thiazolidinediones, dipeptidyl peptidase-4 inhibitors, SGLT2 inhibitors, and GLP-1 receptor agonists, each with distinct efficacy and safety profiles [30-32].

Monotherapy demonstrates reduced durability in progressive T2DM, with declining beta-cell function and multifactorial metabolic disturbances often necessitating treatment escalation [33]. This provides the rationale for combination therapy, which targets complementary mechanisms simultaneously, including insulin resistance, impaired insulin secretion, hepatic glucose production, glucagon dysregulation, and renal glucose reabsorption [34,35]. Outcomes varied according to the specific drug classes used in combination, with more favourable cardiometabolic and weight profiles observed in regimens incorporating SGLT2 inhibitors or GLP-1 receptor agonists, whereas sulfonylurea- or insulin-containing regimens were associated with higher hypoglycemia and weight-related trade-offs [23,36]. Combination regimens may provide additive or synergistic glycemic effects and support broader cardiometabolic risk modification depending on the agents selected [8,37].

**Figure 1:**
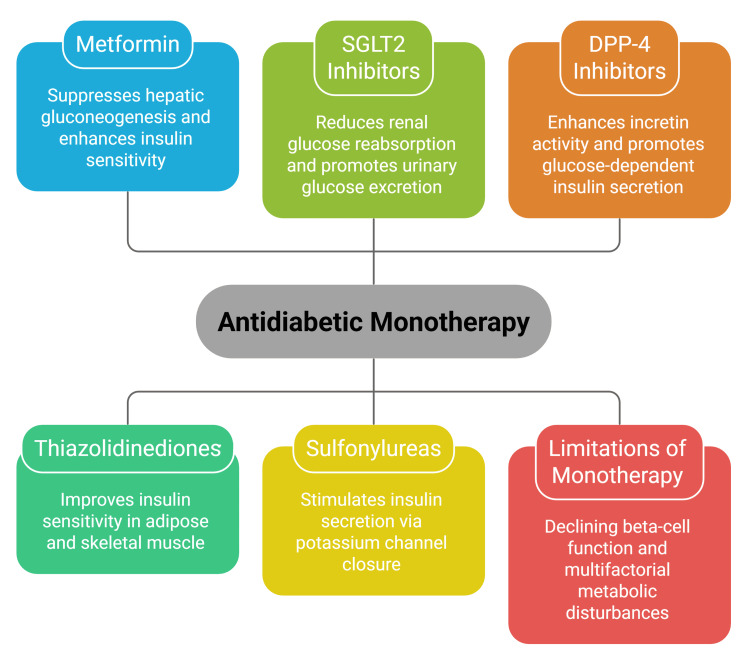
Overview of antidiabetic monotherapy in T2DM T2DM: type 2 diabetes mellitus; SGLT2: sodium-glucose cotransporter 2; DPP-4: dipeptidyl peptidase-4 The figure is an original creation by the authors.

Early combination therapy has been proposed as an alternative to stepwise escalation, particularly in patients with high baseline glycated haemoglobin or high cardiometabolic risk [38]. Fixed-dose combination formulations may reduce pill burden and simplify treatment regimens, potentially improving adherence [18]. Figure 1 summarises major antidiabetic drug classes used as monotherapy and key limitations associated with single-agent strategies in T2DM.

Glycemic control outcomes

Glycemic control remains a primary therapeutic goal in T2DM due to its association with reduced microvascular complications and long-term metabolic stability [39,40]. Comparative analyses demonstrate differences between monotherapy and combination therapy in the magnitude and durability of HbA1c reduction [38]. Monotherapy typically achieves HbA1c reductions depending on baseline glycemic levels and drug class [22,41].

Early Combination Therapy

Combination therapy has been associated with greater HbA1c reductions than monotherapy through complementary mechanisms of action [42]. Reductions exceeding 1.5% are frequently reported with dual regimens, particularly when agents target distinct metabolic pathways [7]. Early use of combination therapy may facilitate more rapid achievement of glycemic targets in patients with higher baseline HbA1c.

Sequential Step-Up Intensification

Stepwise intensification based on glycemic response remains common in clinical practice [38]. Although this approach allows individualised adjustment, delayed escalation may prolong exposure to hyperglycemia and limit long-term glycemic optimisation.

Durability and Stability of Glycemic Control

Durability of glycemic response differs between treatment strategies. Progressive beta-cell dysfunction contributes to declining efficacy with single-agent therapy over time [43]. Combination therapy may mitigate this decline by targeting multiple metabolic mechanisms simultaneously [36]. Regimens incorporating SGLT2 inhibitors or GLP-1 receptor agonists with metformin have demonstrated sustained glucose-lowering effects and reduced need for further escalation in both clinical trials and real-world analyses [14,44]. Table 2 summarises major glycemic outcomes of monotherapy and combination therapy in T2DM.

**Table 2:** Comparative glycemic control outcomes with monotherapy and combination therapy in T2DM HbA1c: glycated haemoglobin; T2DM: type 2 diabetes mellitus; Met: metformin; SU: sulfonylureas; DPP-4i: dipeptidyl peptidase-4 inhibitors; SGLT2i: sodium-glucose cotransporter 2 inhibitors; GLP-1RA: glucagon-like peptide-1 receptor agonists

Therapy type	Mean HbA1c reduction	Fasting glucose effect	Postprandial glucose effect	Glycemic durability	References
Met monotherapy	1-1.5%	Significant reduction	Moderate improvement	Limited in time	[37]
SU monotherapy	1-1.5%	Moderate reduction	Significant improvement	Declines with beta-cell loss	[24]
Met + DPP-4i	1.2-1.8%	Moderate reduction	Significant improvement	Moderate durability	[41]
Met + SGLT2i	1.5-2%	Significant reduction	Moderate improvement	High durability	[29]
Met + GLP-1RA	1.5-2.2%	Moderate reduction	Significant improvement	High durability	[32]

Cardiovascular outcomes

Cardiovascular disease remains the leading cause of morbidity and mortality in T2DM, highlighting the importance of therapeutic strategies that extend beyond glycemic control [42]. Cardiovascular effects of antidiabetic therapies vary by drug class, baseline cardiovascular risk, and disease duration [37]. Comparative evaluation of monotherapy and combination therapy indicates differences in cardiovascular risk modification, particularly when agents with established cardioprotective effects are incorporated into treatment regimens [43].

Cardiovascular Effects of Monotherapy

Monotherapy with certain glucose-lowering agents, particularly metformin, has been associated with favourable cardiovascular outcomes in selected patient populations [27]. In contrast, sulfonylureas and insulin have demonstrated neutral or less consistent cardiovascular outcome profiles, with hypoglycemia risk and weight gain contributing to less favourable cardiometabolic effects [20]. Thiazolidinediones improve insulin sensitivity but may increase heart failure risk due to fluid retention [9].

Cardioprotective Effects of Combination Therapy

Combination therapy incorporating SGLT2 inhibitors and GLP-1 receptor agonists has demonstrated reductions in major adverse cardiovascular events and heart failure hospitalisation [44]. When included within combination regimens, these agents retain cardioprotective properties while contributing additional glucose-lowering effects [24]. Evidence supports their use across diverse patient populations, particularly those with established cardiovascular disease or high cardiometabolic risk [45].

Mechanisms and Real-World Evidence

Selected combination regimens may exert effects beyond glycemic control, including influences on blood pressure, body weight, and metabolic parameters [16]. Observational studies have reported differences in long-term treatment patterns between combination therapy and single-agent strategies in routine clinical practice [11]. Early integration of multi-mechanistic regimens aligns with outcome-oriented management approaches in T2DM [40].

Metabolic and weight-related effects

Body weight and metabolic parameters are clinically relevant in T2DM management due to their association with insulin resistance and cardiovascular risk [26]. Antidiabetic therapies differ in their effects on body weight, lipid metabolism, and blood pressure, influencing therapeutic selection [34]. Metformin is generally associated with weight neutrality or modest weight reduction and has demonstrated favourable effects on lipid parameters in some populations [25,42]. In contrast, sulfonylureas and insulin-based therapies are commonly associated with weight gain, which may adversely affect insulin sensitivity and metabolic control [39]. Thiazolidinediones improve insulin sensitivity but are linked to weight gain and fluid retention, limiting their use in patients with obesity or cardiovascular risk [43].

**Figure 2:**
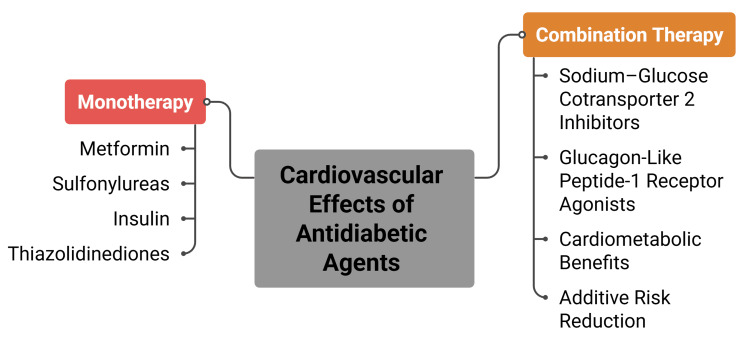
Cardiovascular effects of monotherapy vs. combination therapy in T2DM T2DM: type 2 diabetes mellitus The figure is an original creation by the authors.

Combination therapy allows the integration of agents with complementary metabolic profiles. SGLT2 inhibitors promote weight reduction through urinary glucose excretion and are associated with modest blood pressure decreases [14]. GLP-1 receptor agonists induce weight loss through appetite suppression and delayed gastric emptying and may provide additional lipid benefits [28]. Regimens incorporating these agents may mitigate weight gain observed with insulin secretagogues or insulin therapy and contribute to broader cardiometabolic risk modification [33]. Selected combination regimens have demonstrated favourable effects on triglycerides, high-density lipoprotein cholesterol, and blood pressure, extending benefits beyond glycemic control [8,27]. Figure 2 summarises comparative metabolic considerations of monotherapy and combination therapy in T2DM.

Safety and adverse event profiles

Safety considerations are central to therapeutic decision-making in T2DM due to the chronic nature of treatment and frequent comorbidities [44]. Adverse event profiles vary according to drug class and patient characteristics, with differences observed between monotherapy and combination regimens [38].

Hypoglycemia Risk

Hypoglycemia remains a clinically significant adverse event associated with certain glucose-lowering therapies. Metformin, dipeptidyl peptidase-4 inhibitors, SGLT2 inhibitors, and GLP-1 receptor agonists are associated with low intrinsic hypoglycemia risk due to glucose-dependent mechanisms of action [38]. Sulfonylureas and insulin-based therapies carry a higher hypoglycemia risk, particularly in older adults and those with renal impairment [32]. Combination regimens that include insulin secretagogues or insulin may increase hypoglycemia risk without appropriate dose adjustment [28].

Gastrointestinal Adverse Effects

Gastrointestinal symptoms, including nausea, vomiting, and diarrhoea, are frequently reported with metformin and incretin-based therapies, particularly during initiation and dose escalation [21]. GLP-1 receptor agonists are associated with higher rates of gastrointestinal intolerance compared with dipeptidyl peptidase-4 inhibitors, although gradual titration improves tolerability [34].

Renal and Genitourinary Safety

SGLT2 inhibitors are associated with increased risk of genital mycotic infections and volume depletion, particularly in older individuals and those receiving diuretics [11]. Renal function influences therapeutic selection, as some agents require dose adjustment or avoidance in advanced chronic kidney disease [26].

Cardiovascular and Fluid-Related Concerns

Thiazolidinediones are associated with fluid retention and increased heart failure risk, limiting their use in high-risk populations [37]. Table 3 summarises safety and adverse event profiles of commonly used antidiabetic therapies in T2DM.

**Table 3:** Safety and adverse event profiles of antidiabetic therapies in T2DM T2DM: type 2 diabetes mellitus; Met: metformin; SU: sulfonylureas; DPP-4i: dipeptidyl peptidase-4 inhibitors; SGLT2i: sodium-glucose cotransporter 2 inhibitors; GLP-1RA: glucagon-like peptide-1 receptor agonists

Therapy class	Hypoglycemia risk	Common adverse effects	System-specific concerns	Overall tolerability	References
Met	Low	Gastrointestinal intolerance	Lactic acidosis risk in renal failure	High	[40]
SU	High	Weight gain	Hypoglycemia	Moderate	[36]
DPP-4i	Low	Nasopharyngitis	Rare pancreatitis	High	[43]
SGLT2i	Low	Genital infections	Volume depletion	Moderate-high	[28]
GLP-1RA	Low	Nausea, vomiting	Gastrointestinal intolerance	Moderate	[30]

Patient adherence and treatment persistence

Fixed-dose combination therapy was associated with a lower non-persistence rate of 67.9% compared with 73.4% for loose-dose combination therapy at 12 months [16]. In the same cohort, fixed-dose combination therapy achieved an adherence rate of 57% compared with 50.7% for loose-dose combinations at 12 months based on MPR ≥0.8 criteria [16]. Dipeptidyl peptidase-4 inhibitor monotherapy demonstrated a 12-month persistence rate ranging from 67.4% to 77.2% compared with 57.3% to 73.8% for biguanides at 12 months [27]. In a separate analysis, 12-month persistence was 76.7% for dipeptidyl peptidase-4 inhibitors compared with 68.8% for metformin monotherapy [35].

Metformin demonstrated a median persistence duration of 3.04 years compared with 2.12 years for sulfonylureas over an 11-year observation period [29]. The one-year persistence rate was 70.1% for metformin compared with 64.8% for sulfonylureas [29]. GLP-1 receptor agonists reduced the risk of treatment discontinuation by 15% compared with SGLT2 inhibitors at one year, corresponding to a risk ratio of 0.85 [36]. Self-reported adherence measured using the Morisky Medication Adherence Scale was 61% compared with 79% assessed by direct observation at baseline [42]. High adherence, defined as an MMAS-8 score of 8, was observed in 40% of patients at baseline [45].

Cost-effectiveness and healthcare resource utilisation

Short-Term Costs and Affordability

Empagliflozin was associated with mean total direct healthcare costs of 4274 euros compared with 7009 euros for dipeptidyl peptidase-4 inhibitors over 242-458 days [10]. GLP-1 receptor agonist monotherapy was associated with an average annual cost of 10,771 dollars compared with 13,437 dollars for metformin monotherapy at one year [24]. Metformin monotherapy resulted in an annual per capita cost of 4153 euros compared with 12,890 euros for insulin at one year [39].

Long-Term Costs and Complication Prevention

GLP-1 receptor agonists prevented one case of all-cause mortality for every 57 patients treated compared with insulin over 2.3 years [23]. Metformin plus dipeptidyl peptidase-4 inhibitor therapy was associated with 6.3% higher adjusted overall healthcare costs compared with metformin plus sulfonylurea at one year [35].

Healthcare Resource Utilisation

Empagliflozin reduced the rate of all-cause hospitalisations to 35.45 events per 100 person-years compared with 45.67 events for dipeptidyl peptidase-4 inhibitors over 242-458 days [10]. Adjusted total medical costs were 9189 dollars for GLP-1 receptor agonists compared with 8221 dollars for insulin over 2.3 years [23].

Considerations in Resource-Constrained Settings

Use of the Dario Diabetes Solution resulted in a mean HbA1c reduction of 0.96% compared with 0.73% under standard care at one year [34]. The same intervention generated a net budget impact cost saving of 9,652,498 dollars at one year [34]. Table 4 summarises the comparative clinical outcomes of monotherapy and combination therapy in T2DM.

**Table 4:** Summary comparison of monotherapy and combination therapy in T2DM T2DM: type 2 diabetes mellitus; SGLT2i: sodium-glucose cotransporter 2 inhibitors; GLP-1RA: glucagon-like peptide-1 receptor agonists; SU: sulfonylureas

Domain	Monotherapy	Combination therapy
HbA1c reduction	0.5-1.5%	>1.5% (additive effect)
Durability	Declines with β-cell failure	Greater long-term stability
Weight impact	Neutral or gain (class-dependent)	Weight loss possible (SGLT2i/GLP-1RA)
Hypoglycemia risk	Low–moderate (higher with SU/insulin)	Depends on agents; lower with glucose-dependent drugs
Cardiovascular outcomes	Neutral or modest benefit	Proven risk reduction with SGLT2i/GLP-1RA combinations
Adherence	Generally, higher early persistence due to regimen simplicity	May improve with fixed-dose regimens, but varies by pill burden and tolerability
Cost	Lower short-term medication costs (especially generics)	Higher initial costs with potential downstream savings depending on complication prevention and utilisation

Clinical guidelines and real-world evidence

Clinical practice guidelines inform therapeutic decision-making in T2DM by integrating evidence from randomised trials, observational studies, and expert consensus [40]. International recommendations emphasise personalised treatment selection based on glycemic status, comorbidities, hypoglycemia risk, and overall patient characteristics. Metformin is commonly recommended as first-line therapy in individuals with mild hyperglycemia based on established efficacy and safety data [36]. Combination therapy is advised when glycemic targets are not achieved or when early intensive control is clinically indicated [42]. Recent updates reflect a shift toward outcome-driven treatment algorithms and earlier use of combination strategies in selected high-risk populations [31].

Real-world evidence complements guideline recommendations by evaluating treatment effectiveness across heterogeneous populations and routine care settings [16]. Observational analyses have reported improved glycemic sustainability and reduced treatment escalation with early combination therapy compared with sequential monotherapy strategies [29]. Alignment between guideline-directed care and real-world outcomes supports structured, individualised management approaches in T2DM.

Limitations and future recommendations

Certain limitations merit consideration. Variability in the reporting of adherence metrics, including differences in definitions of medication possession ratio, proportion of days covered, and persistence duration, limits direct comparison across studies. Economic analyses differ in methodology, time horizons, and cost components, restricting uniform interpretation of total healthcare expenditure and budget impact findings.

Future research should prioritise longitudinal comparative analyses incorporating standardised adherence measures and clearly defined economic endpoints. Harmonised reporting of persistence, discontinuation rates, direct medical costs, and healthcare utilisation metrics would enhance comparability across studies.

## Conclusions

Management of T2DM continues to evolve in response to the growing recognition of its complex, progressive, and heterogeneous nature. Comparative evaluation of monotherapy and combination therapy highlights differences across glycemic control, cardiovascular outcomes, metabolic effects, safety, adherence, and economic impact. Monotherapy remains appropriate during early disease stages and in individuals with mild hyperglycemia, offering simplicity, affordability, and favourable tolerability. Combination therapy demonstrates superior and more durable glycemic control through complementary mechanisms of action and provides additional benefits in cardiovascular risk reduction, weight management, and long-term metabolic stability. Overall, optimal management of T2DM requires a balanced, patient-centred approach integrating clinical efficacy, safety, durability, and outcome-oriented treatment selection to achieve sustained metabolic control and improved long-term outcomes.
